# Editorial: Overcoming the Challenges of Herbal Adulteration in a Globalized World

**DOI:** 10.3389/fphar.2021.793616

**Published:** 2021-11-22

**Authors:** Anthony Booker

**Affiliations:** ^1^ Research Group for Optimal Health, School of Life Sciences, College of Liberal Arts and Sciences, University of Westminster, London, United Kingdom; ^2^ Research Group Pharmacognosy and Phytotherapy, School of Pharmacy, University College London, London, United Kingdom

**Keywords:** herbal, supply, adulterant, analytical, Sustainability, quality

The adulteration of plant based products is a global problem and one that cannot be addressed without combined efforts from industry, academia and regulators. This research topic focused on the problems of quality in the broadest sense as well as looking at specific established and new techniques to help improve the detection of sub-standard products. The issues investigated included species authentication, adulteration and contamination, value chains, agricultural practices and sustainability. Authors and reviewers contributed from Asia, the Americas and Europe, with a high percentage of published papers coming from Chinese institutions.

Of the fourteen papers that were accepted for publication, three papers focussed on documenting and highlighting the scale of the problem. Ichim and Booker in their review assessed the scale of the problem globally and found that 37 countries had significant adulteration problems. An opinion piece provided by Palhares et al., the authors described the situation in Brazil and particularly in relation to sustainability and the protection of minority groups and their traditional knowledge. Luo et al. looked specifically at the on-going and increasing problem of heavy metal contamination in Chinese herbal products, something that is often affected by increased industrial pollution of the land and rivers as well as being due to cultivation or collection in areas where they are naturally occurring e.g., in cinnabar containing rock, risking mercury contamination in crops.

There were several papers that explored the value chains of medicinal plants and Kum et al. used Salvia miltiorrhiza as an example of how incorrect species might enter the supply chain and how different processing and storage techniques can affect the end quality of products. Whereas Bi et al. investigated the value chains of Astragalus membranaceus var. mongholicus and assessed the quality in relation to the geographical origin of the material and the effects of using different systems of production and suggested sustainable cultivation as a workable alternative to wild Research Topic. Cultivation may be a viable sustainable option to uncontrolled wild collection, however start up costs and lack of infrastructure can inhibit this process and more governmental and industrial support is needed for these practices to be established long-term.

Other researchers provided new ways to assess the quality of herbal products. Woodley et al. conducted a review of chemical methods and described how mitochondrial activity could be used as a new measure for assessing the biological potency of herbal products and gave detailed methodology on how this could be achieved. Ichim and De Boer described the variation in Ginseng quality and how there were other problems apart from incorrect species, including the wrong part of the plant used and incorrect age of the plant at the time of harvesting (ginseng should be at least 5 years old prior to harvesting in order to obtain the desired amounts of metabolites). There were three papers on shotgun metabarcoding and Yu et al. presented this as a powerful tool for the molecular identification of biological ingredients. This was endorsed by Liu et al. who described how adulterating fungi could be detected in complex herbal mixtures using this technique. Xie et al. showed how shotgun metabarcoding could overcome biased PCR amplification and be used to authenticate herbal mixtures with distinct variation of dosages of individual ingredients. Similarly Yu et al. described specific mini- DNA barcode techniques for use with highly processed herbal products.

Some of the new techniques (or known techniques repurposed) focussed on single plants and Yu et al. described how a method was developed for the analysis of Ophiopogon japonicus that incorporated quantitative microscopy. This allowed for the differentiation of quality within the same species and highlighted the concept of da di (superior quality) herbs in the Chinese herbal supply chain. Ichim et al. also looked at how microscopy could be used as a cost-effective and rapid method to identify impurities within mixtures although with the caveat that it was difficult to use with highly processed materials e.g., extracts. Zhao et al. described vector control quantitative analysis as a new method for analysing complex formulations. A PCR strategy was explained that rapidly generated the integrated DNA fragments needed from multiple targeted species. A technique with much potential for future use within the industry.

It is clear that there are global initiatives in place to improve the detection of poor quality and adulterated material in herbal medicines and there has been advances in many analytical techniques in an effort to overcome the difficulties encountered in analysing complex mixtures of medicinal plants. There appears to be growing emphasis on investigating quality problems along the length of the value chain as opposed to focussing on the finished products and with this comes an increased awareness of good collection and cultivation practices and a greater resolve to tackle issues of sustainability. Overall the papers emphasise the need for effective quality management along the length of the supply chain and the continued application of well known and new techniques to improve herbal quality and safety.

